# Factor Structure and Longitudinal Measurement Invariance of the Demand Control Support Model: An Evidence from the Swedish Longitudinal Occupational Survey of Health (SLOSH)

**DOI:** 10.1371/journal.pone.0070541

**Published:** 2013-08-12

**Authors:** Holendro Singh Chungkham, Michael Ingre, Robert Karasek, Hugo Westerlund, Töres Theorell

**Affiliations:** 1 Stress Research Institute, Stockholm University, Stockholm, Sweden; 2 School of Health and Environment, University of Massachusetts at Lowell, Lowell, Massachusetts, United States of America; University of Rochester, United States of America

## Abstract

**Objectives:**

To examine the factor structure and to evaluate the longitudinal measurement invariance of the demand-control-support questionnaire (DCSQ), using the Swedish Longitudinal Occupational Survey of Health (SLOSH).

**Methods:**

A confirmatory factor analysis (CFA) and multi-group confirmatory factor analysis (MGCFA) models within the framework of structural equation modeling (SEM) have been used to examine the factor structure and invariance across time.

**Results:**

Four factors: psychological demand, skill discretion, decision authority and social support, were confirmed by CFA at baseline, with the best fit obtained by removing the item *repetitive work* of skill discretion. A measurement error correlation (0.42) between *work fast* and *work intensively* for psychological demands was also detected. Acceptable composite reliability measures were obtained except for skill discretion (0.68). The invariance of the same factor structure was established, but caution in comparing mean levels of factors over time is warranted as lack of intercept invariance was evident. However, partial intercept invariance was established for *work intensively*.

**Conclusion:**

Our findings indicate that skill discretion and decision authority represent two distinct constructs in the retained model. However removing the item *repetitive work* along with either *work fast* or *work intensively* would improve model fit. Care should also be taken while making comparisons in the constructs across time. Further research should investigate invariance across occupations or socio-economic classes.

## Introduction

The demand-control model proposed by Karasek [1] defines job strain as a consequence of the combination of high psychological job demands and low job decision latitude, which is a combined measure of skill discretion and decision authority. The job strain model has been successfully used to predict a number of health outcomes, particularly cardiovascular disease [2]–[7]. Later, support at work was added to the model as a dimension that may buffer the effect of high strain on stress-related illness [7].

The original job content questionnaire (JCQ) comprises of 49 items in six dimensions including psychological demands, decision latitude, social support at work and other work characteristics such as physical demands, macro-level decision authority and job insecurity [8]. Several studies have been carried out to validate the JCQ in different sub-groups of workers in Europe [9]–[11], North America [12], [13], Asia [14]–[18] and South America [19].

In 1988, Theorell proposed a 17-item short version of JCQ, the Swedish demand control support questionnaire (DCSQ). This is mostly used in the Scandinavian countries and comprises three dimensions-psychological demands, decision latitude and social support at work [20]. To date there have been five validation studies of DCSQ [21]–[25]. A three-factor solution has been confirmed on a representative sample of Norwegian workers [22]. However, for men in the four occupational groups of International Standard Classification of Occupations (5–9 ISCO major groups) with the highest status, decision latitude dimension fitted better when subdivided into skill discretion and decision authority in a four-factor solution. Two studies [23], [24] examined the psychometric properties of the DCSQ in Brazil using a sample of nursing workers. Skill discretion and decision authority formed two distinct dimensions. This also indicated that the removal of some of the items in further studies might be warranted [23]. From a methodological point of view, there is also a need to look into the interaction between job demands and job control in order to empirically test the theoretical model. But this requires a large sample size [26]. Most of the studies on the demand-control model are based on only moderately large sample sizes. There is a need to look into the feasibility of the model in a nationally representative sample. It is also evident that studies that have used longitudinal research designs have generally been more supportive of the demands-control model as compared to studies using cross-sectional designs [27], [28]. Thus researchers are encouraged to use longitudinal designs wherever possible to test the demands-control model [29]. Kristensen [30] and Van Der Doef and Maes [5] reviewed many of the theoretical criticisms of the model, which could motivate changes to the model to improve its practical value.

There are few studies based on nationally representative longitudinal data examining the factor structure of DCSQ over time. When validating a particular scale, it is important to examine the structural stability of different dimensions. In addition, in order to determine whether DCSQ is structurally stable over time and across different groups, it is necessary to examine the measurement invariance. However, there are some studies which looked into cross-language differential and occupational-differential construct validity of the job content questionnaire using differential item functioning (DIF) approach [31], [32]. They identified cross-language differential item functioning in some of the items across the countries under study. So far there is only one study [33] which uses a measurement invariance test under the confirmatory factor analysis framework. But that study was based on a cross-sectional design. In a seminal review, Vandenberg and Lance [34] described a comprehensive paradigm for evaluating measurement invariance within a confirmatory factor analysis (CFA) framework. To make valid comparisons of mean scores of a particular construct over time it is important that respondents perceived the same underlying meaning of the items, otherwise the similarities or differences may be due to measurement artifacts. If any environmental change (e.g. economic crisis) has happened during a period, this may have an impact on the respondents' perceptions about working conditions thereby leading to invalid comparisons of the underlying construct. In this regard it is important to examine the measurement invariance of the multi-dimensional factor structure of DCSQ. Therefore, the main purpose of this study is to evaluate the factor structure of the demands-control model and to examine the measurement invariance over time using a representative sample of the Swedish working population.

## Data and Methods

The study was approved by the relevant Research Ethics committee (Regionala etikprövningsnämnden i Stockholm: Dnr 2009/1587-31/5). The present study uses data from the Swedish Longitudinal Survey on Health (SLOSH). This is a longitudinal study representative of the Swedish working population in 2003–2005. So far four waves of data collection have been completed (2006, 2008, 2010 and 2012). This study is based on data from the last three waves. These three waves were selected to cover more respondents for longitudinal analysis where the first wave includes only 5141 working individuals. The cross-sectional analysis was based on a total sample of 9756 working individuals from second wave and the longitudinal analysis on 4913 individuals who worked in all the last three waves at the time of survey.

SLOSH uses the Swedish version of DCSQ (Appendix S1). Each item in psychological demands and decision latitude is scored on a Likert-scale ranging from 1 (often) to 4 (never/almost never). Except the two items regarding enough time and repetitive work, all scores are reversed in order to obtain high values for confirmative answers. Social support at work consists of six items, and the response categories are also graded on a four-point Likert-scale ranging from 1 (strongly agree) to 4 (strongly disagree).

### Statistical analyses

Initially, univariate analysis was used to describe the distributions of the items included in the model. Following this, the whole sample was divided into two sub-samples comprising 40% (sub-sample-A) and 60% (sub-sample-B) of the total cases. Sub-sample-A was used for exploring the model based on exploratory factor analysis (EFA) using oblique rotation for correlated factors by retaining factors having eigenvalues greater than one [35], with sub-sample-B used for validating the established model from EFA based on confirmatory factor analysis (CFA) using structural equation modeling framework. The EFA on sub-sample-A was followed by CFA to see the acceptability of factor structure and also for possible model modification.

The most plausible model from sub-sample-A was replicated on the larger sub-sample-B to cross-validate the confirmed the model. This cross-validation approach attempts to minimize sensitivity to sample-specific variation.

The validated model on sub-sample-B was assessed for measurement invariance over time based on full sample in the next stage of analysis. Goodness of fit was evaluated through several fit indices [36]. Root mean square error of approximation (RMSEA) incorporates a penalty function for poor model parsimony, expressed by model degrees of freedom. Values under 0.06 are recommended; whereas values above 0.10 indicate poor fit and that the model should be rejected [37]. The comparative fit index (CFI) represents an incremental fit index comparing the hypothesized model to a more restricted nested baseline model; values above 0.95 indicate good fit [36]. In the initial factor structure examination, modification indices (MI) were also explored in order to identify parameter misfit. This index reflects how much the overall model chi-square would decrease if a constrained parameter was freely estimated. Possible correlations between indicator measurement errors not previously specified in the model under inspection involving values of a modification index equal or more than 10 would further examined, as well as the magnitude of the corresponding expected parameter changes (EPC) for freely estimated parameters [36].

The overall internal consistency of items in the factor structure was tested by calculating composite reliability (CR) with the relaxation of the assumptions of equal common factor loadings and uncorrelated measurement errors posed in Cronbach's alpha. It has already been pointed out that Cronbach's alpha is a lower bound to reliability and tends to give a grossly underestimated value of reliability in most cases [38]. For a particular factor it is calculated as, 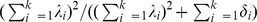, where *λ_i_* is the standardized loading for the *i^th^* item, and *δ_i_*, the corresponding measurement error from the fitted model. Its value lies between 0 and 1 with value ≥0.70 indicate acceptable internal consistency [39]. The 95% confidence intervals for CR were estimated by bootstrapping with 10000 replications [40].

The longitudinal measurement invariance can be evaluated under multiple group confirmatory factor (MGCF) model framework by supplying the mean and covariance structure of the observed variables where the dependence of repeated observations over time was taken into account. This was achieved by modeling each of the latent DCSQ scores at waves II, III and IV as three separate variables nested within individuals and by allowing correlations between the three waves of the survey for the latent factor score and for each item's residual [34], [41], [42]. Details of model framework are given in the web appendix.

Measurement invariance is usually investigated by a series of nested models sequentially with more added restrictions and was tested against the less-constrained model [43]. We used the following tests of measurement invariance: configural invariance [44] to examine the pattern of salient and non-salient loadings, or an equivalent factor structure, across time [34]; metric invariance i.e.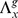 = 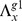 (44) constraining the factor loadings over time to determine whether the expected change in observed values of the indicators per unit change of the construct were equal [34], or that the indicators demonstrated equal relationships with the construct over time.

Since in the present study we have more than one construct, it is of research interest to examine whether variability and the relationships among the constructs are stable across time. This can be done by constraining factor variances and equal factor co-variances to be equal across time. The factor variances represent the dispersion of the latent variables and thus the variability of the construct continua within time. Failure to reject the null hypothesis of equal factor variances indicates that individuals used equivalent ranges of the construct continuum to respond to the indicators reflecting the construct(s) over time. The factor co-variance equivalence can be examined by putting equality in factor co-variances across time. The next test of invariance is the test of equality of unique item variances i.e. 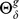 = 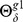 and obtained by constraining like items' uniqueness to be equal between across time. This test has been treated by most researchers as a test for invariant indicator reliabilities across time [45]. The final test that has been taken is the scalar invariance [46] which constrained the intercepts over time/domain i.e. 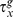 = 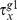 to test whether the observed values of the indicators at any factor value were equivalent across occasions/domains [36], or that differences in means of the indicators were due to differences in the construct [47]. This last test was the most critical step in the procedure because after the demonstration of scalar invariance across time, mean change over time in domains can be attributed to true change in the construct, but establishing this invariance is very infrequent in most research [34], [36].

Except for configural invariance, partial invariance was examined, whenever complete invariance did not hold for a model, by relaxing the equivalence constraint for failing items (i.e. letting them free to vary over time). Partial invariance implies that the parameter under study is invariant for some but not all items. It is an acceptable alternative when complete invariance cannot be reached [48]. The factor-ratio procedure developed by Cheung and Resvold [48] was used to identify non invariant items at the metric and subsequent levels of invariance. An item that is shown to be non-equivalent over time at a specific level of invariance remains unconstrained in the investigation of the next levels of invariance. However, additional research is required to further increase confidence that their procedure is viable [34].

The chi-square difference tests are generally recommended to test measurement invariance by comparing nested models. However, the χ^2^ difference test may also be influenced by sample size [49], thus, a change in CFI between nested models of ≥−0.010 in addition to a change in the RMSEA of ≥0.015 or a change in SRMR of ≥0.030 (for loading invariance) and ≥0.010 (for intercept invariance) is recommended as an appropriate criterion indicating a decrement in fit between models [50]. However, Chen suggested using the change in CFI among the three indices for nested model comparisons as the other two are affected by sample size. But recently a cut-off value of 0.002 for the change in CFI was recommended for lack of invariance [51]. We adopted this criterion for assessing lack of measurement invariance over time. All statistical tests was carried out in *lavaan version 0.5–12* package [52] of *R* [53] with the use of full information maximum likelihood (FIML) estimation with weighted least square adjusted for mean and variance (WLSMV) to account for the ordinal responses of the items included in the model. Under this estimation the difference in χ^2^ does not follow a chi-square distribution, so we use the scaled χ^2^ [54]. In practice, applied researchers do not have much knowledge about the missing data mechanism. In the absence of such knowledge, FIML is a superior method than ad hoc methods, such as listwise deletion, pairwise deletion for dealing with missing data in structural equation models [55].

## Results

Descriptive statistics for all the items included in the model are presented in Table 1. Many of the items showed low to moderate non-normality, except for certain items in all the waves of survey. In all the waves, two items of skill discretion construct i.e. *skill level* and *ingenuity* showed high values of skewness and kurtosis compared to other items in the model.

**Table 1:** Distributions of the items of Demand-Control-Support scale over 3 waves.

Items of constructs	Wave-II in 2008, *N_2_* = 9756				Wave-III in 2010, *N_3_* = 9132				Wave-IV in 2012, *N_3_* = 7325			
	Mean	S.D.	Skew	Kurt	Mean	S.D.	Skew	Kurt	Mean	S.D.	Skew	Kurt
**Psychological demands (PSD)**												
*work fast^R^*	3.05	0.68	−0.45	3.36	2.96	0.71	−0.52	3.51	2.97	0.69	−0.28	1.97
*work intensively^R^*	2.88	0.79	−0.39	2.83	2.71	0.80	−0.34	2.72	2.67	0.80	−0.19	0.19
*work effort^R^*	2.85	0.78	−0.19	2.53	2.71	0.78	−0.18	2.66	2.75	0.77	0.22	2.45
*enough time*	3.03	0.78	−0.41	2.59	2.99	0.82	−0.40	2.49	1.98	0.84	0.71	1.91
*conflicting demands^R^*	2.63	0.76	−0.12	2.66	2.66	0.77	−0.18	2.68	2.68	0.79	0.33	3.35
**Skill discretion (SD)**												
*learning new things^R^*	3.25	0.69	−0.63	3.26	3.24	0.68	−0.64	3.38	3.20	0.68	−0.37	1.65
*skill level^R^*	3.63	0.56	−1.35	4.59	3.62	0.57	−1.33	4.60	3.63	0.56	−1.21	2.82
*ingenuity^R^*	3.50	0.64	−1.07	3.75	3.51	0.63	−1.12	3.97	3.51	0.63	−0.95	1.50
*repetitive work*	3.04	0.82	−0.46	2.50	2.96	0.88	−0.44	2.36	2.02	0.89	0.80	2.31
**Decision authority (DA)**												
*how to do the work^R^*	3.43	0.71	−1.07	3.71	3.33	0.77	−1.04	3.63	3.32	0.76	−0.81	1.20
*what to do at work^R^*	2.95	0.85	−0.38	2.39	2.86	0.91	−0.36	2.28	2.84	0.89	−0.33	−0.67
**Social support at work (SSW)**												
*pleasant atmosphere^R^*	2.77	0.74	−0.38	3.04	2.83	0.74	−0.38	3.06	2.93	0.74	−0.35	0.71
*spirit of unity^R^*	3.05	0.71	−0.52	3.39	3.05	0.72	−0.53	3.32	3.11	0.69	−0.40	1.04
*colleagues support^R^*	3.16	0.67	−0.52	3.52	3.17	0.66	−0.54	3.63	3.22	0.64	−0.50	0.49
*coworkers helpful^R^*	3.06	0.68	−0.49	3.57	3.09	0.67	−0.47	3.49	3.14	0.67	−0.52	0.60
*relationship with superiors^R^*	3.26	0.67	−0.71	3.75	3.28	0.67	−0.72	3.76	3.28	0.68	−0.80	0.89
*relationship with colleagues^R^*	3.38	0.61	−0.59	3.24	3.36	0.64	−0.70	3.48	3.40	0.63	−0.77	0.69

*Note*: *^R^*: reversed items; *N_i_*: sample sizes; S.D: standard deviation; Skew: skewness; Kurt: kurtosis.

### Cross-Sectional EFA followed by CFA Models

The EFA applied on sub-sample-A (n = 3861) of *wave-II* survey showed four clear correlated factors with eigenvalues greater than one with slightly low loadings for the items *conflicting demands* (0.44) of the psychological demands and *repetitive work* (0.36) of the skill discretion in agreement with other studies [e.g. 31]. This suggested suitability of solutions up to four factors. Various alternatives of CFA models were estimated following EFA to examine the most plausible Demand-Control-Support models that fits the data. The results are shown in Table 2. It is evident that the four-factor solution EFA model (Model-I) showed low item loadings for the skill discretion construct, particularly for *repetitive work* (λ = 0.34) with high item measurement error >0.80. This model resulted in poor fit indices (CFI = 0.947; RMSEA = 0.077). Therefore, this model is not acceptable.

**Table 2:** Standardized loadings (*λ_i_*), measurement errors (*δ_i_*), factor correlations (*Φ_ij_*), composite reliability (*ρ_cr_*) and fit indices from competing confirmative factor analysis (CFA) models of the demand-control-support questionnaire based on sub-sample-A.

Items of constructs	Model-I		Model-II		Model-III	
	*λ_i_* *	*δ_i_*	*λ_i_* *	*δ_i_*	*λ_i_* *	*δ_i_*
**Psychological demands (PSD)**						
a. *work fast*	0.69	0.53	0.56	0.68	0.59	0.66
b. *work intensively*	0.69	0.53	0.58	0.67	0.61	0.63
c. *work effort*	0.74	0.45	0.78	0.39	0.83	0.31
d. *enough time*	0.68	0.54	0.70	0.51	0.65	0.58
e. *conflicting demands*	0.53	0.72	0.55	0.70	0.47	0.78
r. pleasant atmosphere	-	-	−0.25	0.45	-	-
Composite reliability for **PSD**	0.73 (0.71, 0.74)		0.73 (0.72, 0.74)		0.68 (0.67, 0.69)	
**Skill discretion (SD)**						
f. *learning new things*	0.56	0.68	0.53	0.72	0.49	0.76
g. *skill level*	0.78	0.39	0.80	0.35	0.82	0.32
h. *ingenuity*	0.78	0.39	0.78	0.40	0.79	0.37
i. *repetitive work*	0.34	0.88	-	-	-	
Composite reliability for **SD**	0.58 (0.56, 0.61)		0.68 (0.67, 0.70)		0.66 (0.63, 0.68)	
**Decision authority (DA)**						
j. *how to do the work*	0.94	0.12	0.94	0.11	0.90	0.18
k. *what to do at work*	0.78	0.41	0.76	0.43	0.79	0.37
Composite reliability for **DA**	0.70 (0.68, 0.73)		0.75 (0.72, 0.78)		0.75 (0.72, 0.78)	
**Social support at work (SSW)**						
l. *pleasant atmosphere*	0.73	0.47	0.63	0.45	-	-
m. *spirit of unity*	0.88	0.23	0.88	0.22	-	-
n. *colleagues support*	0.90	0.19	0.90	0.19	-	-
o. *coworkers help*	0.81	0.35	0.81	0.35	-	-
p. *relationship with superiors*	0.64	0.59	0.64	0.59	-	-
q. *relationship with colleagues*	0.84	0.30	0.84	0.29	-	-
Composite reliability for **SSW**	0.86 (0.85, 0.87)		0.86 (0.85, 0.87)		-	
Item error correlation (item-a_???_item-b)	-		0.42 (0.39, 0.45)		0.39 (0.38, 0.42)	
**Factor correlations**(*Φ_ij_*)						
*Φ_12_* (PSD, SSW)	−0.33 (−0.35, −0.31)		−0.30 (−0.31, −0.28)		-	
*Φ_14_* (PSD, SD)	0.35 (0.33, 0.37)		0.40 (0.38, 0.42)		0.41 (0.40, 0.43)	
*Φ_34_* (DA, SD)	0.41 (0.38, 0.44)		0.38 (0.35, 0.40)		0.38 (0.37, 0.40)	
*Φ_13_* (PSD, DA)	−0.14 (−0.17, −0.11)		−0.15 (−0.17, −0.13)		−0.13 (−0.14, −0.11)	
*Φ_24_* (SSW, SD)	0.16 (0.14, 0.18)		0.18 (0.16, 0.19)		-	
*Φ_23_* (SSW, DA)	0.31 (0.29, 0.34)		0.31 (0.29, 0.34)		-	
**Goodness of fit indices**						
CFI	0.947		0.967		0.970	
RMSEA	0.077 (0.075, 0.080)		0.067 (0.064, 0.069)		0.061 (0.057, 0.066)	

* *p*<0.05; figures in parentheses are 95% confidence intervals for estimates.

At this stage we examined the modification indices (MI) suggested from Model-I to identify the reasons for the lack of model adequacy. The MIs showed that there is a cross-loading of *pleasant atmosphere* on the psychological demands construct, which would decrease the model's chi-square by 330.8, with an expected parameter change (EPC) of −0.25. MIs also showed that an error measurement correlation between *work fast* and *work intensively* would decrease the model's chi-square by 354.5 with an EPC of 0.33. In the initial exploration, we also found that the item-rest correlation of the *repetitive work* item of skill discretion was only 0.27 which is quite low. This shows that this particular item does not fit well with the rest of the items in the particular construct. Based on the low values of loading and item-rest correlation of the item *repetitive work* we tested an alternative four-factor model (Model-II) without the item *repetitive work*, in which the cross-loading of *pleasant atmosphere* on psychological demands (−0.25) and the error measurement correlation between *work fast* and *work intensively* were confirmed (0.43).The model showed a greater improvement over Model-I and was adequate with acceptable fit indices (CFI = 0.967, RMSEA = 0.067), as suggested by Hu and Bentler [36]. The composite reliability (CR) for skill discretion was moderate (0.68), and the correlations among the constructs are all significant at *p*<0.05.

**Figure 1:**
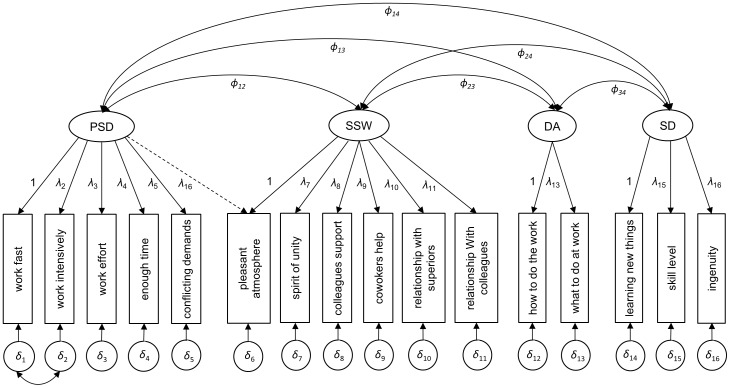
Revised conceptual Demand-Control-Support model.

In the final stage, we fitted a model (Model-III) without the social support at work dimension, by correlating measurement errors of two items of psychological demands found in Model-II to check suitability of the original job strain concept proposed by Karasek. The model shows a good fit with CFI = 0.970; RMSEA = 0.061 and a significant improvement from all earlier models in terms of fit indices. However, these three models are not comparable statistically as their likelihoods are not comparable due to a differing number of factors in the models. Therefore, these final two models are equally good. The preference of one over another should be guided by the research question and the feasibility of the model. There were mixed findings regarding inclusion of social support dimension in the demand-control model [22], [23]. But in a close look at the final two models we found that the loading of the item *learning new things* of skill discretion is relatively low (0.48) in Model-II, although this model is equally good as Model-II. With this recommendation and from the theoretical point of view we retained Model-II for validation and measurement invariance in later stages of analyses. A graphical depiction of our final model is shown in Figure 1.

### Cross-Sectional Model Cross-Validation

The results of the CFA model validated on sub-sample-B are shown in Table 3 (Model-IIa). The model showed an acceptable good fit indices (CFI = 0.970, RMSEA = 0.061). This indicates that the established four-factor DCSQ model is not heavily influenced by sample variation.

**Table 3:** Standardized loadings (*λ_i_*), measurement errors (*δ_i_*), factor correlations (*Φ_ij_*), composite reliability (*ρ_cr_*) and fit indices from validated model of the demand-control-support questionnaire based on sub-sample-B.

Items of constructs	Model-IIa	
	*λ_i_* *	*δ_i_*
**Psychological demands (PSD)**		
a. *work fast*	0.55	0.70
b. *work intensively*	0.56	0.68
c. *work effort*	0.78	0.39
d. *enough time*	0.70	0.52
e. *conflicting demands*	0.56	0.69
r. pleasant atmosphere	−0.27	0.45
Composite reliability for **PSD**	0.73 (0.42, 0.74)	
**Skill discretion (SD)**		
f. *learning new things*	0.57	0.68
g. *skill level*	0.78	0.39
h. *ingenuity*	0.77	0.42
Composite reliability for **SD**	0.69 (0.67, 0.72)	
**Decision authority (DA)**		
j. *how to do the work*	0.91	0.17
k. *what to do at work*	0.77	0.40
Composite reliability for **DA**	0.75 (0.73, 0.77)	
**Social support at work (SSW)**		
l. *pleasant atmosphere*	0.61	0.45
m. *spirit of unity*	0.87	0.24
n. *colleagues support*	0.90	0.20
o. *coworkers help*	0.77	0.40
p. *relationship with superiors*	0.64	0.59
q. *relationship with colleagues*	0.84	0.30
Composite reliability for **SSW**	0.86 (0.85, 0.87)	
Item error correlation (item-a_???_item-b)	0.39 (0.36, 0.41)	
**Factor correlations**(*Φ_ij_*)		
*Φ_12_* (PSD, SSW)	−0.32 (−0.33, −0.31)	
*Φ_14_* (PSD, SD)	0.37 (0.35, 0.38)	
*Φ_34_* (DA, SD)	0.35 (0.33, 0.37)	
*Φ_13_* (PSD, DA)	−0.16 (−0.18, −0.14)	
*Φ_24_* (SSW, SD)	0.21 (0.19, 0.22)	
*Φ_23_* (SSW, DA)	0.29 (0.27, 0.31)	
**Goodness of fit indices**		
CFI	0.970	
RMSEA	0.061 (0.058, 0.063)	

* *p*<0.05; figures in parentheses are 95% confidence intervals for estimates.

### Longitudinal Measurement Invariance of the Demand-Control-Support Questionnaire (DCSQ)

Model-II is the baseline model use for the longitudinal measurement invariance tests. The results of the longitudinal measurement invariance tests are listed in Table 4. The parameter estimation uses full-information maximum likelihood (FIML) by incorporating all individuals who responded in any of the items in either wave (N = 4913). The configural invariance model (Table 4, first line) indicated good fit 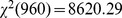, *p*<0.05; CFI = 0.953; RMSEA = 0.044). The primary parameter estimates obtained from the configural invariance are shown in Table 8 (Web Appendix).

**Table 4:** Longitudinal measurement invariance tests of demand-control-support factor model over time [*N* = 4913].

Competing Models	*χ^2^* (*df*)	RMSEA	Δ *χ^2^* *(Δ*df*)	CFI (ΔCFI)
Configural invariance (M1)	10268(960)	0.044	–	0.953
Metric invariance (M2) vs. M1	10380(986)	0.043	58.23(26)	0.954(0.001)
Factor variance invariance (M3) vs. M2	10493(994)	0.042	34.37(8)	0.954(0.000)
Observed error invariance (M4) vs. M3	10969(1002)	0.043	214.82(8)	0.953(−0.001)
Observed intercept invariance (M5) vs. M4	12044(1098)	0.043	1605.00(96)	0.948(−0.005)
Partial intercept invariance (M6) vs. M4	11750(1092)	0.043	1163.10(90)	0.951(−0.002)

*Note:*

* Satorra-Bentler scaled chi-square difference.

After establishing configural invariance over time, the next step is to evaluate whether the relationships between items of a particular construct is the same over time (Table 4, second line). This model also gave a good fit indices (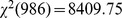, *p*<0.05; CFI = 0.954; RMSEA = 0.043). Compared with the model of configural invariance, this model yields a change in chi-square which is significant at *p*<0.05, however, there was a change of 0.001 in CFI as suggested by Meade [51]. We have thus established metric invariance.

The factor variance equivalence is tested by constraining the variances of the same constructs to be equal across time in addition to having equivalent factor loadings for the corresponding items. This model is also acceptable according to the fit indices (Table 4, M3), showing that over the three time points respondents used equivalent ranges of the demand-control-support construct continua. In comparison to the metric invariance model, the change in CFI is almost negligible. This indicates the invariance of factor variances over time which is logical since we have already established the existence of equal number of conceptual constructs included in the demand-control model over time (i.e. configural test) [34].

Next is a test of the invariance of the unique item variances across time (Table 4, M4). This is undertaken by constraining like items' error variance to be equal across time for the particular construct under consideration. Since it has already been established that the factor variance is invariant across time, the establishment of this test would indicate invariant reliabilities [45]. The uniqueness invariant model was indeed accepted with a small change in CFI and small values of RMSEA and SRMR.

The final test of longitudinal measurement invariance is of scalar invariance, obtained by constraining like items' intercepts to be equal across time separately for each of the constructs included in the model. The results (Table 4, M5) showed an adequate fit with CFI = 0.948. However, this model resulted in a decrement of fit when tested against the uniqueness invariance model (*change in* χ^2^(96) = 1605.00, *p*<0.05; change in CFI = −0.003). To assess which indicators of the demand-control dimensions are responsible for the lack of intercept invariance over time, modification indices from the scalar invariance model (M5) were evaluated along with the factor-ratio test suggested by Cheung and Resvold [48]. This indicated that the thresholds of one of the item of psychological demands construct differed across time: *work intensively (MI = 350)*. It was also evident the largest changes in intercepts over time were in this item of psychological. Therefore, a model (Table 4, M6) with freely estimated thresholds for this item of psychological demands in addition to the restrictions imposed in the previous model (M5) in the form of partial invariance was fitted. This improved the fit considerably compared to M4 (*change in* χ^2^(90) = 1163.10, *p*<0.05; change in CFI = −0.002). Since the partial intercept invariance model (M6) is accepted, we can calculate latent factor means across time. The results indicate that relative to the latent scores at *Wave-II*, the scores on all four sub-dimensions (*psychological demands*, *skill discretion*, *decision authority and social support*) of the Demand-Control-Support Model were significantly higher in *Wave-III* (0.08, 0.03, 0.15 and −0.01) except for *social support*. The respective scores of *psychological demands*, *skill discretion*, *decision authority and social support* in *Wave-IV* relative to *Wave-II* were 0.08, 0.06, 0.16 and −0.07. Thus, the uniqueness invariance model is suggested as the final model of full invariance over time.

Since we have three time points, the omnibus analysis for the measurement invariance may miss some of the between-wave differences. To address this problem we further explored the measurement invariance across each pair of waves. The results are shown in Tables 5–7. The first panel is for the invariance across waves II and III (freely estimating for wave IV), the second for waves II and IV (freely estimating for wave III), and the third for waves III and IV (freely estimating for wave II). We found partial measurement invariance for each of the pairs of waves-II, III and II, IV. But full measurement invariance was established for the pair of waves III and IV. A possible explanation would be the environmental change (e.g. economic crisis in 2008) that may change the workers' perceptions about psychological job demands.

**Table 5:** Longitudinal measurement invariance tests of demand-control-support factor model across 2008 & 2010.

Competing Models	*χ^2^* (*df*)	RMSEA	Δ *χ^2^* *(Δ*df*)	CFI (ΔCFI)
Configural invariance (M1)	10268(960)	0.044	–	0.953
Metric invariance (M21) vs. M1	10331(973)	0.043	33.45(13)	0.953(0.000)
Factor variance invariance (M31) vs. M21	10379(977)	0.043	17.31(4)	0.953(0.000)
Observed error invariance (M41) vs. M31	10666(981)	0.043	67.83(4)	0.953(0.000)
Observed intercept invariance (M51) vs. M41	11229(1029)	0.044	527.08(48)	0.950(−0.003)
Partial intercept invariance (M61) vs. M41	10982(1017)	0.043	476.810(36)	0.951(−0.002)

*Note:*

* Satorra-Bentler scaled chi-square difference.

**Table 6:** Longitudinal measurement invariance tests of demand-control-support factor model across 2008 & 2012.

Competing Models	*χ^2^* (*df*)	RMSEA	Δ *χ^2^* *(Δ*df*)	CFI (ΔCFI)
Metric invariance (M22) vs. M1	10334(973)	0.043	32.71(13)	0.954(0.001)
Factor variance invariance (M32) vs. M22	10431(977)	0.043	28.01(4)	0.954(0.000)
Observed error invariance (M42) vs. M32	10447(981)	0.043	2.31(4)	0.954(0.000)
Observed intercept invariance (M52) vs. M42	11279(1029)	0.043	1179.40(48)	0.950(−0.004)
Partial intercept invariance (M62) vs. M42	10957(1017)	0.043	713.14(36)	0.952(−0.002)

*Note:*

* Satorra-Bentler scaled chi-square difference.

**Table 7:** Longitudinal measurement invariance tests of demand-control-support factor model across 2010 & 2012.

Competing Models	*χ^2^* (*df*)	RMSEA	Δ *χ^2^* *(Δ*df*)	CFI (ΔCFI)
Metric invariance (M23) vs. M1	10307(973)	0.043	21.45(13)	0.954(0.001)
Factor variance invariance (M33) vs. M23	10334(977)	0.042	7.54(4)	0.954(0.000)
Observed error invariance (M43) vs. M33	10756(981)	0.044	94.09(4)	0.952(−0.002)
Observed intercept invariance (M53) vs. M43	10973(1029)	0.043	334.48(48)	0.951(−0.001)

*Note:*

* Satorra-Bentler scaled chi-square difference.

**Table 8:** Unconstrained unstandardized factor loadings, and error variances in the configural invariance model of the DCSQ over time.

Items	*Wave-II*		*Wave-III*		*Wave-IV*	
	Loadings	Item errors	Loadings	Item errors	Loadings	Item errors
**Psychological Demands**						
*Work fast*	1.00	0.66	1.00	0.64	1.00	0.68
*Work intensively*	1.01	0.65	0.95	0.68	1.00	0.68
*Work effort*	1.29	0.43	1.30	0.40	1.39	0.38
*Enough time*	1.13	0.56	1.09	0.58	1.12	0.60
*Conflicting demands*	1.02	0.65	0.96	0.67	1.04	0.66
**Skill Discretion**						
*Learning new things*	1.00	0.70	1.00	0.64	1.00	0.64
*Skill level*	1.37	0.43	1.23	0.46	1.23	0.46
*Ingenuity*	1.46	0.35	1.37	0.34	1.36	0.34
**Decision Authority**						
*How to do the work*	1.00	0.23	1.00	0.16	1.00	0.18
*What to do at work*	0.90	0.37	0.90	0.32	0.89	0.35
**Social Support at Work**						
*Pleasant atmosphere*	1.00	0.44	1.00	0.43	1.00	0.43
*Spirit of unity*	1.41	0.24	1.35	0.22	1.37	0.21
*Colleagues support*	1.44	0.19	1.38	0.18	1.40	0.18
*Coworkers help*	1.23	0.41	1.23	0.35	1.27	0.33
*Relationship with superiors*	1.05	0.58	0.97	0.60	1.04	0.54
*Relationship with colleagues*	1.35	0.29	1.30	0.28	1.33	0.26

As suggested by one reviewer, in addition to examining the measurement invariance on the full sample, we have tested the measurement invariance on the split samples (40∶60%). We found the same pattern of measurement invariance in both subsamples. This confirms that the results from the measurement invariance are not biased by sampling fluctuations.

## Discussion

This study has confirmed a significantly correlated two-factor structure for decision latitude. A study, based on a sample of Brazilian hospital nurses and restaurant workers [23] also found the same factor structure. In another study, Sanne et al. [22] using the same Swedish version of DCSQ as in the present study found a similar factor pattern, but that study was restricted to men in high-status and women in low-status occupation groups. Other validation studies with JCQ have confirmed the same factor structure [14], [56]–[58], [10].

In the present study we got a low loading of the item *repetitive work*, although the relationship with its construct is still significant. However, several JCQ studies found non-significant loadings of *repetitive work* [1], [56], [57], [9], [10], [16], [18], indicating that this item does not go well with the rest of items in the subscale. We found low loadings of the two items *work fast* and *conflicting demands* of the psychological demands construct. This finding is slightly different from other studies, where there were low loadings for *enough time* and *conflicting demands* [1], [57], [9], [59], [10], [13], [16]. However, the low loading of the item *conflicting demands* was also found in one of the studies based on JCQ using European data set [31]. In one study, Karasek et al. [60] found different frequency distributions for *enough time* and *repetitive work* when applied JCQ and DCSQ simultaneously to the same Swedish sample of “Job Stress, Absenteeism and Coronary Heart Disease European Cooperative (JACE)” study in five countries. This may be due to differences in item wordings and/or response options which alter the relationships between the items and constructs and hence the findings related to these items would not be comparable [23].

The present paper also has certain drawbacks. Notably, we did not take into account the heterogeneity of study population with respect to job categories. Of course this will be a very complex data structure under longitudinal set up. A further exploration may be to evaluate the measurement invariance with respect to certain job and demographic characteristics, such as sex, type of job etc. or to see differences between old and new cohorts in the latest wave of SLOSH.

The present study also has certain strengths. First it is based on a large representative sample of the entire working population in Sweden. Second, this study used longitudinal design which can capture the stability or change in factor structure of the demand-control model under the framework of structural equation modeling, which is the first of its kind in this area of research.

A moderate correlation between skill discretion and decision authority (Model-II) is in agreement with findings from the Brazilian study using DCSQ [23], but Karasek et al. [60] found a higher correlation between the two constructs for JCQ compared to DCSQ. This difference may reflect the difference in the number of items in the two scales [23].

In validation studies of JCQ and DCSQ, the role played by the social support construct is not well defined, although there is a well-established interaction effects with other constructs [7]. As far as the decision regarding the inclusion of this construct in the model the experience has been mixed. Most studies of JCQ included this construct in the factor analysis [14], [9], [59], [10], [16]–[18], while others excluded it [57], [61], [13]. However, two of the studies using DCSQ in different contexts, one by Sanne et al. [22] and another by Hökerberg et al. [23], showed high item loadings of the social support construct. However, the first study included the construct and the second one concluded that the construct should be excluded from the final model of the factor structure. In our present study, we also found high item loadings for the social support dimension, but models with the construct did not provide support for good-fitting models. The small cross-loading of the *pleasant atmosphere* item of social support subscale on psychological demands seems to indicate that the atmosphere at the workplace may have some impact on the perception of the demands at work. We made an attempt to fit an independent model based on the original model proposed by Karasek without the social support dimension. The fitted model also showed good acceptable fit, but some of the items resulted low loadings. Therefore, inclusion or exclusion of the dimension should also be guided by the research question. With this and from the theoretical point of view we retained the model with social support.

Psychological demands at work and control over the work have been and are used extensively as the primary framework for both researchers and practitioners to understand the impact of the psychosocial work environment on health. However, as different cohorts of individuals enter the working population, there may be shifts in the perceptions of the demands at work and the control over the work tasks. In addition, it is important to determine whether the demand-control model established for a working population remains a useful measure as individuals experience significant changes in work culture. In addition, it is also important not to assume that tools consistently measure the same constructs over time. In order to precisely measure the true change and inter-individual differences, it is important to examine if the established demand-control model exhibits measurement equivalence across time.

Therefore, tests of longitudinal measurement invariance are pre-requisites for understanding whether changes in demands-control level over time reflect true changes in exposure or rather changes in the assessment or structure of the demands-control. In this paper, the result of configural invariance test indicated that the factor structure depicted in Figure 1 was equivalent over the four-year period studied and across the three time points separated by two years. The metric invariance model which tested whether the items included in factors established equal relationships with their own factors over time and the model indicates that the relationships are comparable over the four years in each correlated but separate factor included in the demand-control model. We also concluded that the variance in the different dimensions of the demand-control model was equivalent over time. This shows that individuals used an equivalent range of the demand-control constructs continuum to respond to the indicators reflecting the respective constructs. The inter-relationships among the constructs of the demand-control model were also stable over time. However, the final test of invariance i.e. intercept equivalence resulted in a decrement in fit when compared to the item-error equivalence model.

The lack of intercept invariance suggests that the zero points of some of the latent scores of each of the factors included in the model are not the same over time. We found one item of the psychological demands is responsible for lack of intercept invariance. Therefore, although the mean levels of each of factors of demand-control model may remain unchanged over time, the indicators of the corresponding factors may fluctuate. Thus, the interpretation of change in the mean scores of the psychological demands of DCSQ over time may be misleading because observed changes in the means of factors may arise due to changes in the measurement properties of the indicators which is not entirely due to change in true factor means. An individual may have an unchanged true level of the psychological demands at work over time, yet the observed values of the indicator may lead researchers to believe that the psychological demands have increased/decreased. This lack of full invariance in the psychological demands over time may also be from the differences in items threshold between the second and subsequent waves. This phenomenon where data collected in the second wave of a survey differ from those collected in the following waves is not uncommon [39]. It may signal some sort of panel effect [62] or Hawthorne effect where respondents become familiar with the survey procedure following the second wave and modify their behavior or attitude accordingly. The pairwise measurement invariance tests suggested full invariance of the DCSQ over the period 2010–2012 (i.e. between waves III and IV). However, partial invariance was established in each of other pairs of waves. The lack of invariance in the psychological demands over time may be partly due to the financial crisis at the time of wave II. At that time most of the jobs are unstable which may influence the respondents' perceptions about psychological demands at work. However, after certain period when the economy becomes more or less stable, their perceptions about job demands may continue from that stable period. This was indicated by the invariance of the demands between 2010 and 2012, but partial invariance in each of the pairs 2008, 2010 and 2008, 2012 from the pairwise invariance tests. But to establish intercept invariance depends on the research question also. If the main purpose of the research is to compare mean levels of underlying constructs then one need to establish intercept invariance, otherwise care should be taken while comparing the mean levels over time.

## Conclusion

The present study has confirmed existence of four correlated dimensions by splitting decision latitude into skill discretion and decision authority representing the demand-control-support model. Weak correlations among the dimensions were found with decision authority was negatively correlated with psychological demands. In conclusion consistent with other findings the item *repetitive work* should be removed from skill discretion and the two items *work fast* and *work intensively* psychological demand are duplicated and could be removed one of them. Also the established factor structure of the demand-control-support model is fairly stable over time. However, differences in observed mean levels of the psychological demands do not reflect true differences in the constructs measured by the observed variables. This has warranted either to remove the non-invariant item (*work intensively*) in further development of the scale or not to use in the mean comparisons of the construct over time.

## Supporting Information

Appendix S1 **Demand control support questionnaire (in English and Swedish).**
(DOCX)
