# Supplementary material for: Factor Structure and Longitudinal Measurement Invariance of the Demand Control Support Model: An Evidence from the Swedish Longitudinal Occupational Survey of Health (SLOSH)
Source: PLoS One. 2013 Aug 12;8(8):e70541. doi: 10.1371/journal.pone.0070541 (PMC3741382; doi:10.1371/journal.pone.0070541)
Supplement: Appendix S1 — Demand control support questionnaire (in English and Swedish). (DOCX) [file pone.0070541.s001.docx]

**Appendix S1**

Demand control support questionnaire (English wording).

| Psychological demands | |
| --- | --- |
| 1. | Do you have to work (very) fast? (*work fast*) |
| 2. | Do you have to work very intensively? (*work intensively*) |
| 3. | Does your work demand too much effort? (*work effort*) |
| 4. | Do you have enough time to do everything? (*enough time*) |
| 5. | Does your work often involve conflicting demands? (*conflicting demands*) |
| Decision latitude | |
| Skill discretion | |
| 6. | Do you have the possibility of learning new things through your work? (*learning new things*) |
| 7. | Does your work demand a high level of skill or expertise? (*skill level*) |
| 8. | Does your work require ingenuity? (*ingenuity*) |
| 9. | Do you have to do the same thing over and over again? (*repetitive work*) |
| Decision authority | |
| 10. | Do you have a choice in deciding how you do your work? (*how to do the work*) |
| 11. | Do you have a choice in deciding what you do at work? (*what to do at work*) |
| Social support at work | |
| 12. | There is a calm and pleasant atmosphere where I work (*pleasant atmosphere*) |
| 13. | There is a good spirit of unity (*spirit of unity*) |
| 14. | My colleagues are there for me (*colleagues support*) |
| 15. | People understand that I can have a bad day (*cowokers helpful*) |
| 16. | I get on well with my superiors (*relationship with superiors*) |
| 17. | I get on well with my colleagues (*relationship with colleagues*) |

Demand control support questionnaire (Swedish wording).

| Psykologiska krav | |
| --- | --- |
| 1. | Kräver ditt arbete att du arbetar (mycket) fort? (*arbeta snabbt*) |
| 2. | Kräver ditt arbete att du arbetar mycket hårt? (*arbeta intensivt*) |
| 3. | Kräver ditt arbete en för stor arbetsinsats? (*arbesinsats*) |
| 4. | Har du tillräckligt med tid för att hinna med arbetsuppgifterna? (*tillräckligt med tid*) |
| 5. | Förekommer det ofta motstridiga krav i ditt arbete? (*motstridiga krav*) |
| Handlingsutrymme | |
| Skicklighet diskretion | |
| 6. | Får du lära dig nya saker i ditt arbete? (*lära sig nya saker*) |
| 7. | Kräver ditt arbete skicklighet? (*kompetensnivå*) |
| 8. | Kräver ditt arbete påhittighet? (*uppfinningsrikedom*) |
| 9. | Innebär ditt arbete att man gör samma sak om och om igen? (*repetitivt arbete*) |
| Beslut myndighet | |
| 10. | Har du frihet att bestämma hur ditt arbete ska utföras? (*hur man gör arbetet*) |
| 11. | Har du frihet att bestämma vad som ska utföras i ditt arbete? (*vad man ska göra på jobbet*) |
| Socialt stöd på arbetet | |
| 12. | Det är en lugn och behaglig stämning på min arbetsplats (*trevlig atmosfär*) |
| 13. | Det är god sammanhällning (*anda av enighet*) |
| 14. | Mina arbetskamrater staller upp för mig (*kolleger stöder*) |
| 15. | Man har förståelse för att jag kan ha en dålig dag (*medarbetare hjälp*) |
| 16. | Jag kommer bra överens med mina överordnade (*relation med överordnade*) |
| 17. | Jag trivs med mina arbetskamrater (*relation med kollegor*) |

Longitudinal multiple group confirmatory factor (MGCF) model framework:

The measurement relationships between the *k* items in the *g*^th^ time point (*g* = 1, 2, 3) under confirmatory factor model fitted to the data can be represented as (Jöreskog &Sörbom, 1996),

$X_{k}^{g}=\tau_{k}^{g}+\Lambda_{k}^{g}\xi^{g}+\delta_{k}^{g}$ …………… (1)

where, $X_{k}^{g}$ refers to the vector of items making the composite measure, $\Lambda_{k}^{g}$ is the matrix of loadings relating $X_{k}^{g}$ to the vector of constructs $\xi^{g}$, $\tau_{k}^{g}$ indicates the vector of regression intercepts, and $\delta_{k}^{g}$ represents the vector of item measurement errors.

The covariance equation from (1) can be written as,

$\Sigma^{g}=\Lambda_{X}^{g}\Phi^{g}\Lambda_{X}^{g'}+\Theta_{\delta}^{g}$ …………… (2)

where, $\Sigma^{g}$ is the matrix of variances and co-variances among the *K* items in the *g*th time point, $\Lambda_{X}^{g}$ is the matrix of items’ factor loadings on $\xi^{g}$, $\Phi^{g}$ involves variances and co-variances among the constructs $\xi^{g}$, and $\Theta_{\delta}^{g}$ is the matrix of unique variances.

**Table 4**. Unconstrained unstandardized factor loadings, and error variances in the configural invariance model of the DCSQ over time.

| Items | *Wave-II* | |  | *Wave-III* | |  | *Wave-IV* | |
| --- | --- | --- | --- | --- | --- | --- | --- | --- |
|  | Loadings | Item errors |  | Loadings | Item errors |  | Loadings | Item errors |
| **Psychological Demands** | |  |  |  |  |  |  |  |
| *Work fast* | 1.00 | 0.66 |  | 1.00 | 0.64 |  | 1.00 | 0.68 |
| *Work intensively* | 1.01 | 0.65 |  | 0.95 | 0.68 |  | 1.00 | 0.68 |
| *Work effort* | 1.29 | 0.43 |  | 1.30 | 0.40 |  | 1.39 | 0.38 |
| *Enough time* | 1.13 | 0.56 |  | 1.09 | 0.58 |  | 1.12 | 0.60 |
| *Conflicting demands* | 1.02 | 0.65 |  | 0.96 | 0.67 |  | 1.04 | 0.66 |
| **Skill Discretion** |  |  |  |  |  |  |  |  |
| *Learning new things* | 1.00 | 0.70 |  | 1.00 | 0.64 |  | 1.00 | 0.64 |
| *Skill level* | 1.37 | 0.43 |  | 1.23 | 0.46 |  | 1.23 | 0.46 |
| *Ingenuity* | 1.46 | 0.35 |  | 1.37 | 0.34 |  | 1.36 | 0.34 |
| **Decision Authority** |  |  |  |  |  |  |  |  |
| *How to do the work* | 1.00 | 0.23 |  | 1.00 | 0.16 |  | 1.00 | 0.18 |
| *What to do at work* | 0.90 | 0.37 |  | 0.90 | 0.32 |  | 0.89 | 0.35 |
| **Social Support at Work** |  |  |  |  |  |  |  |  |
| *Pleasant atmosphere* | 1.00 | 0.44 |  | 1.00 | 0.43 |  | 1.00 | 0.43 |
| *Spirit of unity* | 1.41 | 0.24 |  | 1.35 | 0.22 |  | 1.37 | 0.21 |
| *Colleagues support* | 1.44 | 0.19 |  | 1.38 | 0.18 |  | 1.40 | 0.18 |
| *Coworkers help* | 1.23 | 0.41 |  | 1.23 | 0.35 |  | 1.27 | 0.33 |
| *Relationship with superiors* | 1.05 | 0.58 |  | 0.97 | 0.60 |  | 1.04 | 0.54 |
| *Relationship with colleagues* | 1.35 | 0.29 |  | 1.30 | 0.28 |  | 1.33 | 0.26 |
